# Supplementary material for: Improving case-detection of wasting among under-five-year-old children in Ethiopia: A secondary analysis of community-based surveys in humanitarian settings
Source: PLOS Glob Public Health. 2026 Mar 27;6(3):e0005952. doi: 10.1371/journal.pgph.0005952 (PMC13029798; doi:10.1371/journal.pgph.0005952)
Supplement: Attachment — Submitted filename: Point by Point Responses_PLOSGlobalHealth.docx [file pgph.0005952.s001.docx]

**Point by Point Responses**

| Editorial | Responses |
| --- | --- |
| Please send a completed 'Competing Interests' statement, including any COIs declared by your co-authors. If you have no competing interests to declare, please state "The authors have declared that no competing interests exist". | Stated under the declaration section |
| Please amend your detailed Financial Disclosure statement. This is published with the article. It must therefore be completed in full sentences and contain the exact wording you wish to be published | Amended |
| Please provide separate figure files in .tif or .eps format and remove the embedded figures from the manuscript file. | Provided as separate figures and removed from the manuscript |
| In the online submission form, you indicated that “Data will be provided by the corresponding authors upon reasonable request” | Revised as follows: “The data that support the findings of this study are available from Ethiopia Disaster Risk Management Commission (RDRMC) and Ethiopia Nutrition Coordination Unit (ENCU) but restrictions apply to the availability of these data, which were used under license for the current study, and so are not publicly available. Data are, however, available from the authors upon reasonable request and with permission of EDRMC and ENCU” |

| Reviewer 1 comments | Responses |
| --- | --- |
| WHO defines Wasting as WFH<-3SD score and or MUAC <12.5 cm. Ideally MUAC performance should have been checked for whole GAM identified by both criteria. Authors have compared MUAC vs WFH. | Thank you for the comment. Already GAM, SAM and MAM defined based on WHZ and MUAAC under data processing section lines 169-172. |
| Please check References 19& 20. They both are same. | Thank you for picking this. References revised. |

| Reviewer 2 comments | Responses |
| --- | --- |
| This is well written and well conducted analysis that is relevant to an on-going discussion about the use of MUAC only programs in the community. Additionally, the Ethiopian ministry is very proactive about wasting management programs and so this paper may have policy impact. | Thank you for the comments |
| The paper’s framing and take homes are a bit confusing. You acknowledge that WHO recommends MUAC+WHZ to define SAM, and your conclusion is that this is correct. However the analysis looking at MUAC’s ability to predict WHZ, and you treat WHZ as the gold standard for SAM diagnosis. I think the whole results needs to be framed as MUAC ability to predict WHZ-SAM not as SAM generally. This is not incorrect in your discussion of false positives. In Table 10 you give the False positive rate of a MUAC cut off of 115mm for diagnosis SAM, but all children below 115mm have SAM! This actually only requires a small change – say WHZ-SAM not SAM alone. A similar issue can be raised about all your diagnostic accuracy statistics. For example, the sensitivity of MUAC to detect GAM should be MUAC+/(MUAC+ + WHZ+), but you are giving MUAC+/WHZ+. I agree with your approach, but I just think you need change the terminology your results to say WHZ-GAM and WHZ-SAM. | Thank you for the comment. We agree with framing the terms as SAM-WHZ or GAM-WHZ rather than SAM or GAM. The results section has been revised accordingly to incorporate these terms. |
| In the discussion, line 494, you say WHZ “overestimates prevalence compared to MUAC”. This is the opposite of the above issue – WHZ cannot overestimate because it is SAM. Both MUAC and WHZ are SAM, WHZ alone can only underestimate. Again, a small tweak is needed I think the word “overestimates” is the issue. I think that just saying WHZ estimates are higher than MUAC is sufficient. | Thank you for the comment and the statement revised as per the comment. |
| Conclusion line 577, you say MUAC in two area had “extremely high false positive” even at normal cutoffs. That is not true because MUAC counts SAM. Again it is nuance in how you frame the results that could lead to people incorrectly thinking MUAC is of no value. | Thank you for picking this. The statement revised as follow “Although Somali and Gambella exhibited very high false-negative rates at the standard MUAC threshold, this does not imply that MUAC lacks programmatic value. MUAC remains an effective tool for identifying many severely malnourished children and is widely used for community-level screening due to its simplicity, low cost, and feasibility. The observed variation highlights the need for context-specific strategies and complementary approaches, rather than replacing MUAC” |
| You mention in the introduction that discordant children, those who are MUAC+ WHZ- are still at risk. I think it is important to repeat this in the discussion. There is a risk that your analysis is misinterpreted to imply only WHZ matters. You do not conclude that, but it is important that readers are reminded that discordant children are still at risk. | Thank you for your comment. A paragraph added under discussion section (see lines 528-533). |
| The regional observation that you observe is very interesting and your interpretation, particularly the discussion of stunting and different body compositions is very insightful. One critique maybe that you’ve left room for readers to think that there may be regional variation in the accuracy of measurements – i.e. poor training in some regions or surveys. You only have one sentence in methods saying training was standardized. Is it possible to give more details to reassure readers this variation across regions is not about training differences? | Thank for your comment. Detailed added on the training. See lines 153-158. |
| You mention that the SMART flags exclude very plausible measurements in the methods and limitations. Can you not just turn those flags off as a sensitivity analysis? | We conducted a sensitivity analysis to assess the impact of SMART flags on GAM estimates. The results show that the difference is minimal: 12.9% without flags versus 12.5% with flags, indicating that turning off the flags does not materially change the overall conclusion. However, we retained SMART flags in the final analysis because they are part of internationally recognized quality control standards. The limitation was also revised as “In addition, the use of SMART flags, which exclude measurements deemed statistically implausible, may have resulted in the omission of a small number of biologically plausible cases. However, sensitivity analysis showed that the impact of these exclusions on GAM prevalence by WHZ was minimal (12.9% without flags vs. 12.5% with flags), and the overall conclusions remain unchanged” |
| You conclude that MUAC cutoffs should vary by region. Scientificall that might make sense, but programmatically is that a good idea? It sounds like a nightmare. I think like you should add a caveat that the programtic feasibility of such variation is unclear. | Thank you for your comment. A caveat added see lines 588 -589. |
| What happened to children with oedema in your analysis, were they excluded? | Cases of bilateral pitting edema were not included in estimated prevalence of wasting by WHZ or MUAC; edema cases were relatively rare in all surveys, representing approximately 0.05% of the total cases. Also mentioned under data processing section. See lines 187-188 |
| The “MUAC Diagnostic Performance” heading is repeat. This may be deliberate, but the sections do appear to be distinct. | Thank you for your comment. The heading revised as “MUAC Optimal Threshold” |

| Reviewer 3 comments | Responses |
| --- | --- |
| Line 97: Add spacing between the statements and their corresponding references. Do this throughout the manuscript. | Thank you for your comments. Space added throughout the manuscript. |
| Line 112: mid-upper MUAC? | Corrected |
| Line 113-114: Include the full reference | Reference added and the statement revised |
| Line 122-123: Explain why MUAC is more sensitive in detecting malnutrition among younger and female children compared with older and male children. | Explained. See lines 124-127 |
| Line 136: Define GAM | Defined. See lines 113-114 |
| Line 151-153: Describe how children’s physical measurements were taken to ensure accuracy and consistency, including the types of anthropometric instruments used, their measurement precision and calibration | Described. See lines 150-160. |
| Line 167: Report the number of missing or incomplete anthropometric records, if any were identified. | Reported. See lines 188-190 |
| Line 178: Define HAZ and WAZ. | Defined |
| Line 207-208: Explain how Youden’s index was calculated and specify which index value was used to identify the optimal MUAC cutoffs (the higher YI?). | Explained. See lines 227-229. |
| Line 207: Ensure that the 95% confidence interval for the AUC is included in the results section. | Removed from the method section |
| Line 208-210: Describe how sensitivity, specificity, positive predictive value, negative predictive value, accuracy, and likelihood ratios (LR positive and LR negative) were calculated/defined. | Defined. See lines 231-241. |
| Line 244: In Table 1, add a separate row showing the total number of surveys and children instead of listing the total number of children under the Age (months) variable. | Row added |
| Line 217-219: Include information on participant consent and their rights during data collection. | This study used previously published, anonymized secondary data collected under ethical informed consent during the original survey. No additional consent was required. Also mentioned under ethics statement section. |
| Line 246: n (%)? | Corrected |
| Figure 1-5: Remove the plot titles at the top of each figure because the titles are already provided in the captions | Removed. |
| Line 287-292: Do not repeat numbers that are already reported in the table. | Revised |
| Table 4: Specify in the table footnote what the Kappa statistic represents. Is it measuring agreement between MUAC and WHZ for identifying MAM, normal status, or SAM? | Footnote provided |
| Line 337: Replace the repetitive subtitle with something like “MUAC Diagnostic Accuracy Measures.” | Revised |
| Line 449-450: Clarify whether the MUAC cutoff of 139 mm applies to the entire sample or only to older children. | Clarified |
| Line 464-465: Explain what these numerous instances are by giving some examples of situations in which MUAC and WHZ classify children differently. | Explained under introduction section. See lines 124-128 |
| Line 467-475: Clarify whether the variation in MUAC–WHZ correlations across Ethiopian regions is due to differences in setting, population characteristics, dietary behaviors, or the fact that the two methods detect different aspects of body composition, | Clarified see lines 579-586 |
| Line 487-489: Explain some of the key contextual factors that may influence the association between MUAC and WHZ | Already explained. See lines 502-514. |
| Line 501-512: Excellent. Integrate these justifications into the earlier paragraphs discussing the correlation or association between the two methods. | Already integrated |
